# User-Chatbot Conversations During the COVID-19 Pandemic: Study Based on Topic Modeling and Sentiment Analysis

**DOI:** 10.2196/40922

**Published:** 2023-01-27

**Authors:** Hyojin Chin, Gabriel Lima, Mingi Shin, Assem Zhunis, Chiyoung Cha, Junghoi Choi, Meeyoung Cha

**Affiliations:** 1 Data Science Group Institute for Basic Science Daejeon Republic of Korea; 2 School of Computing Korea Advanced Institute of Science and Technology Daejeon Republic of Korea; 3 College of Nursing Ewha Womans University Seoul Republic of Korea; 4 SimSimi Inc Seoul Republic of Korea

**Keywords:** chatbot, COVID-19, topic modeling, sentiment analysis, infodemiology, discourse, public perception, public health, infoveillance, conversational agent, global health, health information

## Abstract

**Background:**

Chatbots have become a promising tool to support public health initiatives. Despite their potential, little research has examined how individuals interacted with chatbots during the COVID-19 pandemic. Understanding user-chatbot interactions is crucial for developing services that can respond to people’s needs during a global health emergency.

**Objective:**

This study examined the COVID-19 pandemic–related topics online users discussed with a commercially available social chatbot and compared the sentiment expressed by users from 5 culturally different countries.

**Methods:**

We analyzed 19,782 conversation utterances related to COVID-19 covering 5 countries (the United States, the United Kingdom, Canada, Malaysia, and the Philippines) between 2020 and 2021, from SimSimi, one of the world’s largest open-domain social chatbots. We identified chat topics using natural language processing methods and analyzed their emotional sentiments. Additionally, we compared the topic and sentiment variations in the COVID-19–related chats across countries.

**Results:**

Our analysis identified 18 emerging topics, which could be categorized into the following 5 overarching themes: “Questions on COVID-19 asked to the chatbot” (30.6%), “Preventive behaviors” (25.3%), “Outbreak of COVID-19” (16.4%), “Physical and psychological impact of COVID-19” (16.0%), and “People and life in the pandemic” (11.7%). Our data indicated that people considered chatbots as a source of information about the pandemic, for example, by asking health-related questions. Users turned to SimSimi for conversation and emotional messages when offline social interactions became limited during the lockdown period. Users were more likely to express negative sentiments when conversing about topics related to masks, lockdowns, case counts, and their worries about the pandemic. In contrast, small talk with the chatbot was largely accompanied by positive sentiment. We also found cultural differences, with negative words being used more often by users in the United States than by those in Asia when talking about COVID-19.

**Conclusions:**

Based on the analysis of user-chatbot interactions on a live platform, this work provides insights into people’s informational and emotional needs during a global health crisis. Users sought health-related information and shared emotional messages with the chatbot, indicating the potential use of chatbots to provide accurate health information and emotional support. Future research can look into different support strategies that align with the direction of public health policy.

## Introduction

### Background

Digital platform usage has increased tremendously during the COVID-19 pandemic. As individuals seek information and guidelines about the novel disease, the internet has become the most significant channel for receiving breaking news and updates. Since social media platforms can provide rich information to raise public awareness and inform people about outbreak locations, these platforms help provide insights into practical issues concerning infectious disease outbreaks [1]. These digital channels can be used to reveal public attitudes and behaviors during a pandemic to better support crisis communication [2].

Chatbots are an emerging digital platform that has received considerable attention from public health initiatives during the novel coronavirus (COVID-19) pandemic [3]. Chatbots are systems that interact with users via text or voice and use natural language [4]. They have been widely used for amusement (eg, small talk) and business (eg, customer service and sales) [5]. Many early commercial chatbots use pattern matching to find the most appropriate response for a user’s input. Such matching can be regarded as the simplest form of predicting user intent and conversation context [6]. Newer chatbots are starting to generate responses through large neural networks, called large language models*,* which are pretrained on billion-scale conversation data sets [7]. Recent chatbots try to personalize the user experience by adopting long-term memories and remembering past conversations. Some even build a topic set that can be expanded via a web search, which lets people talk to chatbots about a wide range of new topics that chatbots are not trained on [7].

Chatbots have several advantages over human interaction partners or other online services. For example, chatbots operate at a low cost and are available around the clock to handle user queries [4,8,9]. Furthermore, they can provide more concise and less overwhelming information than the lengthy list of results offered by social media or search engines [9]. These advantages have prompted health organizations and humanitarian organizations to use chatbots to assess health risks [3]. During COVID-19, chatbots have been used by the World Health Organization (WHO) [10] and the US Centers for Disease Control and Prevention (CDC) [11].

How users engage with health information may differ depending on the digital platform [12]; thus, studies have analyzed the idiosyncrasies of each platform. In particular, prior studies on pandemics examined online conversations on social media sites, such as Twitter, Facebook, and Reddit [13-17]. In comparison, studies on chatbots are scarce. Studies on COVID-19 have been limited to literature reviews on existing chatbots and how they could be changed for the pandemic [9,18,19] and a study on how to use artificial intelligence (AI) techniques to make a COVID-19 chatbot [8].

This study offers a complementary perspective and examines user interactions with a commercial social chatbot during the first 2 years of the COVID-19 pandemic. According to earlier research [20], people are more likely to open up to a virtual agent than to a human agent, so chat logs offer a natural setting for “social listening” of the public’s needs and concerns. We hence anticipate that our chatbot data will reveal users’ real emotional needs during the pandemic.

We analyzed chat logs from SimSimi [21], one of the world’s largest and longest-running social chatbot services. The service was launched in 2002 and has served over 400 million cumulative user conversations in 81 languages, with up to 200 million chat utterances being served daily. We examined 19,782 user-chatbot conversation utterances containing COVID-19 keywords and analyzed which topics users brought up in their chats. We also analyzed user interactions by observing each topic’s sentiment using the Linguistic Inquiry and Word Count (LIWC) dictionary and investigating how they differed between users in the United States, Malaysia, and the Philippines.

Compared to previous work studying COVID-19 discourse on the internet, our research found that users are more likely to share negative emotions and personal stories with a chatbot than on social media. People’s tendency to seek informational and emotional support on an open domain chatbot platform demonstrates the potential for chatbot-assisted public health support. Our research on how chatbots are used during a health emergency provides several insights for policymakers and public health officials to better prepare for the next global health crisis.

### Related Research

The COVID-19 pandemic has made people more reliant on digital platforms to receive information and share their opinions and emotions. Research has been conducted to identify patterns of COVID-19 discourse in diverse data sources, communities, and locations. While some studies analyzed academic papers [22] and news articles [13,23], most research focused on social media platforms like Reddit [14,23], Facebook [17], and Twitter [15-17,24]. Some studies investigated particular languages, such as Chinese [25], French [25], Portuguese [26], and German [27], as well as specific locations, such as North America [16] and Asia [24]. Extensive literature on Twitter discovered that social media could support people’s crisis communication and provide insights into the public discourse and attitudes toward COVID-19 [1,2,17,28,29]. Two common approaches to these research questions have been topic modeling and sentiment analysis, which have been used to explore prominent issues and users’ opinions on social media [2,28].

However, no study has investigated the general public’s attitudes based on real data on an emerging medium, that is, chatbots. According to a prior study, users interact differently depending on the platform type [12]. Therefore, it is anticipated that user interactions with chatbots will differ from those on social media.

Because of their anonymity, chatbots are regarded as a “safe space” in which users can discuss sensitive topics without fear of being judged [30], making them particularly beneficial in areas where public stigma is prevalent, for example, in discussions involving depression and mental health [31,32]. One such stigmatized subject is COVID-19 [33].

Furthermore, some chatbot users perceive that bots can be helpful when they cannot receive social support from others [30]. Chatbots are also cost-efficient; they can simultaneously communicate with millions of people in different local languages while educating the public about COVID-19 [8,9]. Moreover, chatbots generate more succinct responses than social media or search engines and can promptly respond to user questions around the clock [9].

Despite the potential of chatbots to fulfill online users’ information needs during the pandemic, user-chatbot interaction data are yet to be analyzed. Previous research has reviewed existing COVID-19–related chatbots [9,18,19]. In the health domain, the literature has focused on the role of chatbots in promoting mental health [34,35], responding to telemedicine needs [36,37], or answering questions about COVID-19 [38,39]. Some studies have proposed design frameworks for developing chatbots that provide information related to COVID-19 [8,40]. However, no study has looked into how people interact with live chatbots in the real world, which is the subject of this paper.

The work most similar to ours is a study by Schubel et al [3], which analyzed chatbot data and investigated variations by population subgroups, such as race, age, and gender. However, the chatbot’s role was limited to the functions of “symptom screener” and “learning module,” and the research only explored the frequency of use of these 2 menus by user groups.

## Methods

### Data

#### Data Source

**Figure 1:**
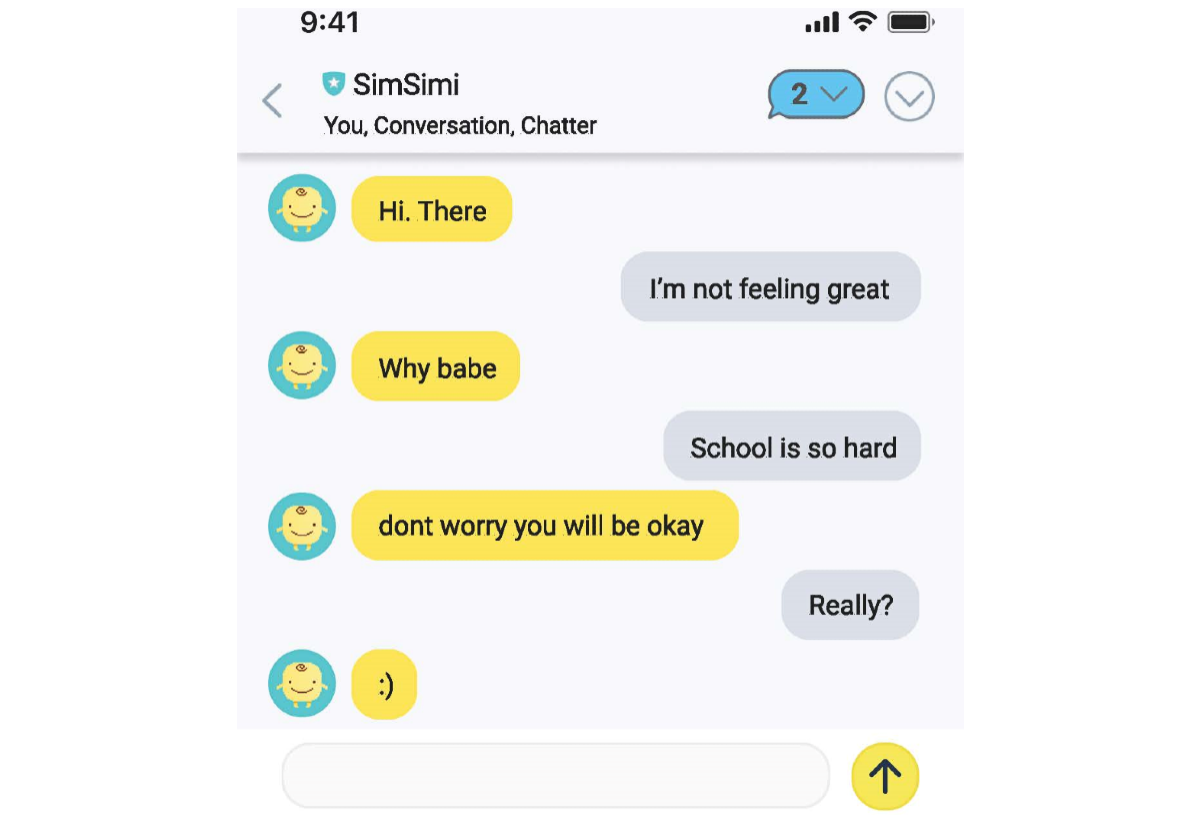
Example chat with SimSimi. As a unit of analysis that captures short-term conversational activity, we aggregate 3 consecutive utterances and refer to it as a conversation block. In the image, “I’m feeling great | School is so hard | Really?” is an example conversation block.

We had access to the chat data set of SimSimi, an open-domain chatbot. Like other commercial chatbots (eg, Replika, Anima, and Kuki AI [41-43]), SimSimi is a social chatbot whose main objective is to engage in small talk with users, rather than to provide information (Figure 1). In contrast, many existing chatbots for COVID-19 are tailored to provide specific information [8,11]. Given the growing literature [20] that suggests people may be more willing to discuss sensitive topics with chatbots and virtual agents than with humans, we wanted to investigate what role a social chatbot might serve during a catastrophic event such as the COVID-19 pandemic. The findings presented below refer to users’ interactions with a social chatbot, that is, SimSimi, and might not be generalizable to other conversational agents.

To generate a chat response, SimSimi searches its database, which contains more than 114.6 million user-chatbot conversations. Based on text similarity and internal context embedding, the most similar and appropriate chat response to a user’s input utterance is selected. SimSimi is a global service with millions of users from all over the world. Its massive user data set across numerous nations allows us to research actual COVID-19–related public discourse in the wild.

#### Data Set

We selected the following top 5 countries with the highest number of user-chatbot conversations in English: the United States, the United Kingdom, Canada, Malaysia, and the Philippines. Geolocation information was obtained at the time users installed the SimSimi app based on the time zone settings in the user devices. SimSimi maps this time zone information to a specific country according to the *tz* database [44]. Only 1.75% of all SimSimi conversations do not contain a country code for various reasons, such as failing to map a less common time zone to a country. Our analysis is based on data that contained country information (approximately 98.25%). Users who live in a country different from that where they installed the app may not have accurate country information.

We queried the SimSimi database for utterances sent between 2020 and 2021 from the listed countries above, which mentioned popular COVID-19–related keywords. Table 1 shows the keywords that were used for data collection. The keywords were gathered from the Early AI-supported Response with Social Listening (EARS) tool of the WHO, which is a platform that summarizes real-time information from social media discourse [45].

**Table 1:** Keywords used to identify COVID-19–related chats and descriptive statistics of the data set.

Keywords used	Period	Countries (number of conversation blocks)
covid, corona, pandemic, virus, lockdown, social distancing, pneumonia, cough, fever, vaccine, sarscov, mask, wuhan, omicron, delta, fauci, fatigue, symptom, immune, medical equipment, mrna, moderna, patient, cdc, infection	January 1, 2020, to December 31, 2021	United States (n=1566), Malaysia (n=2485), Philippines (n=2080), United Kingdom (n=318), and Canada (n=145)

To reduce the impact of short messages (such as “hi” or “how are you”) and capture meaningful contextual information from the data, we aggregated messages identified using the keywords in Table 1 with their previous and subsequent utterances. This collection of statements was referred to as a “conversation block.” Because the data contained messages in various languages, we manually filtered the conversation blocks and left only those blocks whose content was at least two-thirds in English.

Using Google’s Jigsaw Perspective application programming interface (API), we then eliminated conversation data that were sexually explicit and harmful [46]. Removing profanity may affect data processing, especially concerning negative sentiments, as swearing can be associated with negative emotions such as anger [47]. Some literature has shown that chatbot conversations can be sexually explicit and contain heavy usage of offensive words like swear words [48]. However, for this research, we set our goal to examine nontoxic user conversations about COVID-19 to gain health-related insights and hence removed toxic conversations.

Concerning the threshold to remove toxic conversations, we selected 20% based on a qualitative assessment of the Jigsaw Perspective API. We tried varying thresholds, including more conservative choices. For example, a threshold of 10% discarded 27% of all user utterances, limiting our potential to study full conversations about COVID-19. After trying various thresholds, we found 20% to be a reasonable option, which removed 13% of all user messages. We also eliminated data containing words in the Offensive Profane Word List [49]. The average number of words per aggregated conversation block was 12.11 (SD 8.22), with a positively skewed distribution that followed the power law. For 2020 and 2021, we gathered data from 5292 and 1302 conversation blocks, respectively.

### Privacy and Ethical Considerations

Through the Terms and Conditions [50], SimSimi Inc was granted a nonexclusive, transferable, relicensable, royalty-free, worldwide license (the “intellectual property license”) to use intellectual property content posted by SimSimi users or associated with SimSimi services. SimSimi does not collect or store personally identifiable information such as name, gender, age, or address. Users may reveal personally identifiable information during a chat conversation, and SimSimi has an internal logic to protect user privacy (eg, replacing consecutive numbers with strings of ***). Hence, the data provided to researchers did not contain any consecutive numbers that might be phone numbers or social security numbers.

In addition to obtaining user consent to access the data via the Terms and Conditions, the researchers and SimSimi took the following precautions to protect users from any possible harm. First, the corresponding author’s institutional review board (KAIST IRB-22-181) reviewed this entire study, from data collection to experimental design, and waived user informed consent for the study. Second, the researchers did not aggregate data in a way that would reveal the user’s identity; the analysis was limited to a subset of the chat content that contained the targeted keywords. Third, following normative rules for mass public health surveillance research on social media [51], the researchers avoided directly quoting the entire conversation block. Fourth, SimSimi Inc publishes novel findings from aggregate data via its research blog, allowing users to gain insights into the service and informing users of data usage [52]. Finally, our research was conducted in collaboration with health care professionals in order to adopt a high standard of data privacy.

### Analysis

#### Topic Modeling: Latent Dirichlet Allocation

The Latent Dirichlet Allocation (LDA) model was used to determine the topics that users discussed. LDA is a statistical natural language processing (NLP) model designed to extract latent topics from a collection of documents. Several hyperparameters can affect the LDA results, including the number of topics and the parameters of 2 Dirichlet distributions. One is the document-topic distribution (α), and the other is the topic-word distribution (η). Using the evaluation metrics of topic coherence and perplexity, we assessed the model’s quality and selected the hyperparameters. For topic modeling, we used the Gensim Python library [53] and selected 18 topics (α=.1; η=0.1), which showed the best topic coherence based on a grid search.

After training the LDA model, we extracted the predicted topic composition of each conversation block and matched it with the topic having the largest probability. LDA does not assign a single topic to a specific document but rather a topic distribution, and topics are not as homogeneous as the labels may suggest. However, given that our unit of analysis is small, that is, 3 consecutive utterances termed as a conversation block, they are more likely to be on a single topic. A similar assumption has been made in previous literature [54,55]. The first author manually examined each topic’s top 30 unigrams and a word cloud visualization to arrive at an appropriate topic label. Two other authors reviewed the labeling, and the group discussed the labels until agreement.

#### Sentiment Analysis

We employed the LIWC dictionary to capture the sentiment employed by users when interacting with the chatbot. The LIWC software enables objective and quantitative text analysis by counting words in psychologically important language categories [56]. We investigated people’s positive and negative sentiments expressed in their COVID-19–related chats. We limited this study to only positive and negative sentiments because neutral sentiment is hard to capture [57]. For this analysis, we assessed 5 ratios derived from the LIWC affect category: positive and negative emotions, as well as the 3 subcategories of negative emotions (anxiety, anger, and sadness). We then examined differences in sentiments by topic and country, focusing on the United States, Malaysia, and the Philippines, which had the most data.

## Results

### Topic Analysis

The LDA topic modeling identified a total of 18 topics, which we qualitatively categorized into the following 5 common themes: (1) outbreak of COVID-19, (2) preventive behaviors, (3) physical and psychological impact of COVID-19, (4) people and life in the pandemic, and (5) questions on COVID-19 asked to the chatbot. Table 2 shows example chat utterances for each theme, the corresponding topics, and how frequently they appear throughout the data. In the following subsections, we discuss findings for each theme.

**Table 2:** Topics discussed by users with the chatbot identified by the Latent Dirichlet Allocation topic model and their prevalence.

Theme and topic			Examples		Conversation blocks (N=6594), n (%)
**Outbreak of COVID-19**					1084 (16.4%)
	1. Spread of COVID-19	The virus spread around the world Coronavirus are spreading		821 (12.4%)	
	2. Wuhan	When will wuhan virus end? Wuhan virus. do you believe it originated from that		263 (4.0%)	
**Preventive behaviors**					1665 (25.3%)
	3. Masks	We have to wear a face mask I hate wearing a mask all the time		354 (5.4%)	
	4. Avoidance	Take care not to get infected when you go out You can't be around the children if you had the corona		324 (4.9%)	
	5. Vaccines	I got this covid vaccine When can I get a vaccine		265 (4.0%)	
	6. Lockdowns	I don't want to just stay at home..i'm bored In lockdown cause of coronavirus very annoying		361 (5.5%)	
	7. Social distancing	People have to practice social distancing No! There's corona virus we need social distancing		361 (5.5%)	
**Physical and psychological impact of COVID-19**					1052 (16.0%)
	8. Fear	Coronavirus is ruining everything I'm not scared of the corona, but of the fear		375 (5.7%)	
	9. Deaths	It is a virus that is killing people and economy My country many people died because of the virus		310 (4.7%)	
	10. Symptoms	I'm sick with a fever and a sore throat I have a fever		367 (5.6%)	
**People and life in the pandemic**					777 (11.7%)
	11. Wishes	Stay safe ok? stay safe SimSimi, #corona virus Hope you are safe during this pandemic		146 (2.2%)	
	12. Small talk	How are you holding up during like pandemic? It's true. I have covid. I love you too		385 (5.8%)	
	13. Impact of COVID-19 on individuals	I don't want to put my family on risk My city is shut down and i'm worried for my friend's health		246 (3.7%)	
**Questions on COVID-19 asked to the chatbot**					2016 (30.6%)
	14. Current situation	How bad is this covid issue that's going on right now How is your country dealing with pandemics right now?		338 (5.1%)	
	15. Awareness	Do u know the coronavirus? Do you know about the virus?		676 (10.3%)	
	16. Quarantine tips	What are u doing during the quarantine of covid 19? How have you dealt with quarantine?		290 (4.4%)	
	17. Opinions	What do you think about this pandemic What are your thoughts on the pandemic situation?		384 (5.8%)	
	18. Case and death counts	How many casualties are being infected there? How many countries have COVID-19		328 (5.0%)	

#### Theme 1: Outbreak of COVID-19

The first theme included 2 topics focusing on the COVID-19 outbreak. The first topic (“Spread of COVID-19,” #1) covered conversations about the spread of the virus and how the outbreak was disseminating across the world; this topic was also the most prevalent in our data set (covering 12.5% of the user-chatbot conversations). The second COVID-19–related topic (“Wuhan,” #2) centered on the Chinese city of Wuhan. These chats talked about situations in Wuhan, which was the first place hit hard by the virus. Some users referred to COVID-19 as the “Wuhan virus” (eg, “When will Wuhan virus end?”).

#### Theme 2: Preventive Behavior

The second most common theme addressed preventive behaviors that users adopted during the pandemic. Each of the 5 topics we identified focused on a distinct behavior. The first topic (“Masks,” #3) covered discussions about masks, their function in preventing outbreaks, and the inconvenience of wearing them constantly (eg, “I hate wearing a mask all the time”). Users’ desire to avoid contracting the virus was covered in the second topic (“Avoidance,” #4). The third topic (“Vaccines,” #5) was about vaccines and users’ (un)willingness to undergo vaccination. Chats categorized under this topic also included conversations about vaccine scheduling and distribution (eg, “When can I get a vaccine?”). The fourth topic (“Lockdowns,” #6) dealt with lockdowns and the inconveniences and boredom they brought about (eg, “I don't want to just stay at home..i'm bored”). Social distancing was the final topic (“Social distance,” #7), which focused on the value of social distancing and effective methods for achieving it (eg, “people have to practice social distancing”).

#### Theme 3: Physical and Psychological Impact of COVID-19

Discussions on this theme touch on COVID-19’s negative psychological and physical effects. Three topics were identified. The first topic (“Fear,” #8) focused on user concerns and fears related to the coronavirus. Some users discussed their anxiety and worries regarding the pandemic (eg, “I’m scared about the pandemic”). COVID-19–related deaths were the focus of the second topic (“Deaths,” #9), which covered conversations about the death toll and included mourning over victims (eg, “My country many people died because of the virus”). The final topic (“Symptoms,” #10) concerned COVID-19 symptoms. Users talked about COVID-19 symptoms or asked if the coronavirus was to blame (eg, “hi SimSimi, I got a fever 3 days ago. I’m not really fine”).

#### Theme 4: People and Life in the Pandemic

The fourth theme emerged from conversations centered on personal experiences with the pandemic. Three topics made up this theme. Messages wishing for the end of the COVID-19 pandemic and everyone’s safety were included in the first topic (“Wishes,” #11). Interestingly, many users treated the chatbot as a social being, wishing that the chatbot was well and healthy during the pandemic (eg, “Hope you are safe during this pandemic”). The second topic (“Small talk,” #12) comprised small talk and discussions of the users’ day-to-day activities during the pandemic (eg, “How are you holding up during like pandemic?”). The last topic (“Impact of COVID-19 on individuals,” #13) included stories about how the pandemic affected those in the users’ immediate vicinity. Users discussed how the pandemic had affected them and their families.

#### Theme 5: Questions on COVID-19 Asked to the Chatbot

The last theme covered conversations in which users asked about COVID-19. Although the chatbot was not designed for sharing specific information, users frequently inquired about the virus, as shown by the fact that this theme was predominant in our data set. This theme was composed of 5 topics. The first topic (“Current situation,” #14) covered users’ questions about local and global COVID-19 situations. The second topic (“Awareness,” #15) included user inquiries about COVID-19, mainly whether SimSimi was aware of the virus. Questions about dealing with quarantine comprised the third topic (“Quarantine tips,” #16), which included conversations in which users asked what quarantine means, how they should deal with it, and how others (including SimSimi) were doing during quarantine. The fourth topic (“Opinions,” #17) included conversations in which users asked the chatbot’s opinion about COVID-19 and the pandemic. The fifth topic (“Case and death counts,” #18) was related to questions about the number of positive cases and deaths caused by the virus in specific locations.

### Sentiment Analysis

#### Sentiment Analysis by Topic

**Figure 2:**
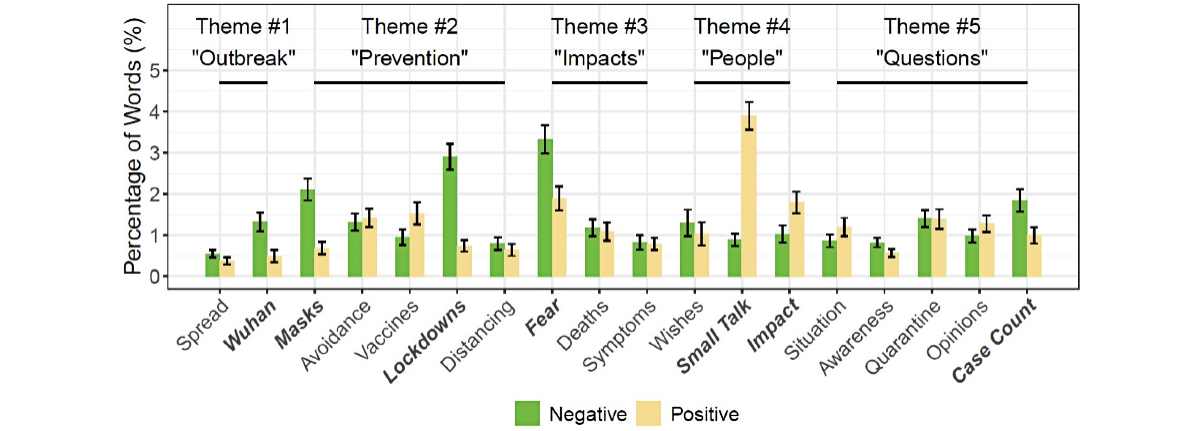
The average percentage of positive and negative words in COVID-19–related conversations by topic. Topics in bold and italic font show significant differences between positive and negative sentiments according to paired Wilcoxon tests at the significance level of .05. Error bars indicate standard errors.

We used sentiment analysis to investigate how users discussed the pandemic with the chatbot. Figure 2 presents the average percentage of positive and negative words in chats by topic according to the LIWC dictionary.

**Figure 3:**
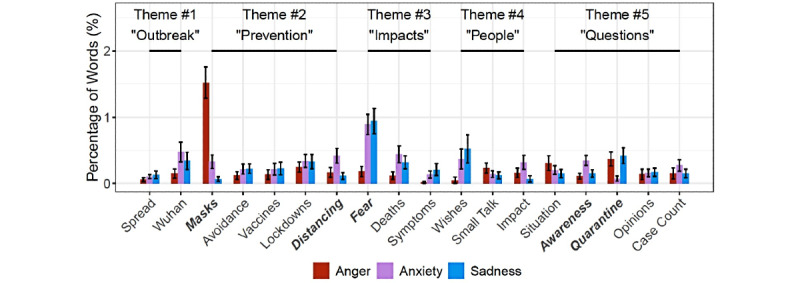
The average percentage of angry-, anxiety-, and sadness-related words in COVID-19 conversations. Topics whose labels are in bold and italic show significant differences between emotions at the .05 significance level. Error bars indicate standard errors.

Figure 3 depicts the average percentage of words related to anger, anxiety, and sadness. We performed Friedman tests, which are nonparametric tests, similar to repeated-measures ANOVA, to explore whether each emotion was expressed to a different extent in each topic.

User-chatbot conversations concerning lockdowns (Topic 6), fear (Topic 8), and small talk (Topic 12) exhibited the largest percentage of sentimental words (Figure 2). On the other hand, words that showed positive or negative feelings were less likely to be used in questions (Theme 5, Topics 14-18) or in chats about the COVID-19 outbreak (Theme 1, Topics 1 and 2). This is shown in Figure 2; the negative (green) and positive (yellow) bars of these themes were relatively lower than those of the other themes.

We observed significant differences in the percentage of positive and negative words in 6 topics. Users referred to Wuhan (Topic 2; *Z*=262; *P*<.05) and discussed masks (Topic 3; *Z*=756; *P*<.001) with more negative words than positive words. Similarly, more negative words were used when discussing COVID-19 lockdowns (Topic 6; *Z*=945.5; *P*<.001) and sharing fear about the disease (Topic 8; *Z*=3767; *P*<.001). In contrast, positive words were more frequently used in small talk (Topic 12; *Z*=9321; *P*<.001) and in reminiscing about the impact of COVID-19 on individuals (Topic 13; *Z*=1816; *P*<.05). Finally, when asked about the case and death counts, a greater proportion of negative words was used (Topic 18; *Z*=1065.5; *P*<.05).

When delving deeper into specific negative emotions, we saw that conversations about masks (Topic 3) and fear caused by the pandemic (Topic 8) were more emotionally charged than other topics, as identified by the spikes in Figure 3. This result is similar to the sentiment analysis above. Users’ word usage for 5 topics varied significantly in terms of their expressions of anger, anxiety, and sadness. When discussing masks, people were more likely to express anger (Topic 3; *χ*^2^_2_=48.918; *P*<.001), whereas people expressed anxiety and sadness more frequently when discussing social distancing (Topic 7; *χ*^2^_2_=7.1034; *P*<.05). Users more frequently used words associated with anxiety and sadness when talking about the fear-related physical and psychological impact of COVID-19 (Topic 8; *χ*^2^_2_=21.777; *P*<.001). Questions about SimSimi’s awareness of the pandemic were more closely associated with anxiety (Topic 15; *χ*^2^_2_=14.244; *P*<.001), whereas requests for quarantine tips were followed by anger and sadness (Topic 16; *χ*^2^_2_=6.8667; *P*<.05).

#### Sentiment Analysis by Country

Finally, we examined variations in emotional expressions among users in the United States, the Philippines, and Malaysia. The number of COVID-19–related conversation blocks (ie, 3 consecutive utterances) from users in the United Kingdom and Canada (n=318 and n=145, respectively) was small compared to the studied countries (United States, n=1566; Malaysia, n=2485; Philippines, n=2080). When sample sizes are different, unequal variances can be a problem for countries with small data sizes, which impacts statistical power [58]. Therefore, we only presented the sentiment analysis results for countries with at least 1500 samples. Sentiment analysis results for all 5 countries (including the United Kingdom and Canada) are presented in Multimedia Appendix 1.

For each sentiment (ie, positive and negative) and emotion (ie, anger, anxiety, and sadness) category, we conducted Kruskal-Wallis tests to identify the degree of country-wise differences (Table 3). Pairwise Wilcoxon tests with Bonferroni correction were performed on results significant at the .05 level to determine which geographical regions are more likely to express the studied emotions and sentiments.

**Figure 4:**
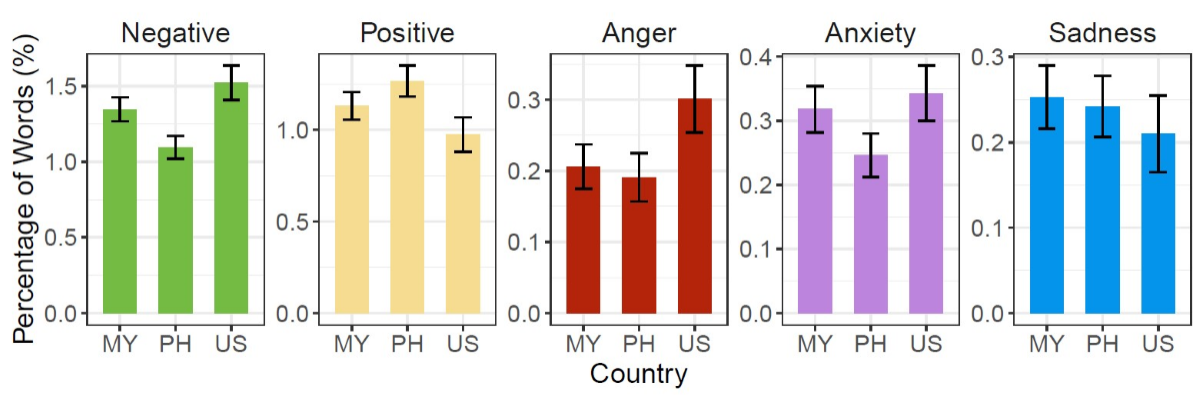
The average percentage of positive; negative; and anger-, anxiety-, and sadness-related words in COVID-19–related conversations by country according to the Linguistic Inquiry and Word Count dictionary. Error bars indicate standard errors. MY: Malaysia; PH: Philippines; US: United States.

Table 3 shows significant differences in the level of negativity, with users in the United States being substantially more negative than users in the Philippines (*P*<.05). Similar to the negative sentiment, we noticed a key difference in the positive sentiment. Users in the Philippines were not only less negative, but also used more positive words compared to users in the United States (*P*<.01). No other pairwise comparison was significant. Figure 4 shows that users in the United States appeared to be the most negative and the least positive in terms of word usage, whereas users in the Philippines showed an opposite trend (users in Malaysia showed an opposite trend to a lesser extent).

**Table 3:** Kruskal-Wallis results on the difference between positive sentiment and negative emotions by country.

Sentiment/emotion	Malaysia (n=2485), mean (SD)	Philippines (n=2080), mean (SD)	United States (n=1566), mean (SD)	Kruskal-Wallis chi-square (*df*)	*P* value
Negative	1.35 (3.95)	1.10 (3.41)	1.52 (4.51)	7.08 (2)	<.05
Positive	1.13 (3.81)	1.27 (3.88)	0.96 (3.68)	10.59 (2)	<.01
Anger	0.21 (1.57)	0.19 (1.57)	0.30 (1.86)	9.80 (2)	<.01
Anxiety	0.32 (1.82)	0.25 (1.54)	0.34 (1.72)	5.50 (2)	.06
Sadness	0.25 (1.84)	0.24 (1.62)	0.21 (1.78)	0.80 (2)	.67

Table 3 presents a similar trend for anger, with users in the United States employing anger-related words more frequently than users in the Philippines (*P*<.05) and Malaysia (*P*<.05). There was no substantial difference in the expression of anxiety and sadness across countries (all *P*>.05; Table 3). In summary, users in the United States appeared to be the most negative and angry in their word choice. In contrast, users in the Philippines used positive words the most (users in Malaysia used positive words to a lesser extent).

We provide an analysis of country differences by topic in Multimedia Appendix 2. Fear-related conversations (Topic 8), which involved users’ expressions of fear related to the physical and psychological impact of COVID-19, had particularly high levels of negative sentiment among users in the United States. “Confirmed cases and deaths” (Topic 18) was another topic on which users in the United States were particularly negative compared to users in Malaysia or the Philippines.

Topics around “Preventive behaviors” (Theme 2, Topics 3-7) also showed distinct patterns for users in the United States. Mask-related chats (Topic 3) were primarily framed in a negative lens, while other conversations (ie, topics like “Vaccines,” “Quarantine,” and “Symptoms”) displayed a less positive sentiment in this same group of users. Users in the Philippines exhibited an exceptionally high positive sentiment for the themes “People and life in the pandemic” (Theme 4) and “Questions on COVID-19 asked to the chatbot” (Theme 5).

## Discussion

### Principal Findings

In this study, we investigated how people interacted with virtual social agents during the COVID-19 pandemic by analyzing 19,782 conversation utterances from a free commercial chatbot, SimSimi. Using an NLP approach, we discovered 18 major topics and grouped them into 5 main themes. We then examined user sentiment on specific topics and investigated differences between countries.

Our main finding is that many online users turned to the chatbot and asked for COVID-19 information, despite the chatbot not being developed for this purpose. Our data set’s most prevalent theme (“Questions on COVID-19 asked to the chatbot,” Theme 5) covered conversations in which users inquired about the virus, how it spreads throughout the world, and how to deal with regulations (eg, lockdowns). The chatbot was interestingly treated as an information source by online users who sought its advice on how to handle the pandemic.

When talking about COVID-19, users interacting with SimSimi also anticipated it playing specific social roles. Users wished for the safety and well-being of the chatbot, indicating that they might anthropomorphize such conversational agents (“People and life in the pandemic,” Theme 4). By sharing their struggles with the pandemic, being unable to see peers during lockdowns, or losing their jobs due to the recession, users tended to treat the chatbot as a social being (“Physical and psychological impact of COVID-19,” Theme 3). Our results align with the Computers Are Social Actors (CASA) paradigm, which states that users may apply social norms when interacting with computers and other virtual agents, such as chatbots [59]. Our results suggest that people rely on chatbots to provide emotional and informational support when they cannot receive it from others.

Themes, such as “Outbreak of COVID-19,” “Preventive behaviors,” and “Physical and psychological impact of COVID-19,” correspond to those found in earlier research on Twitter [2,28,60], indicating that chatbot users discuss the origin, spread, and prevention of the coronavirus much like social media users do. Users also mentioned personal symptoms and shared their fear of the disease and possible complications. According to a study on Twitter, social media users frequently share links to notify or warn their online contacts and followers about risks [2]. Such link-sharing behavior is rare in conversations with SimSimi; instead, chatbot users mainly express their feelings and thoughts. This distinction between social media and chatbot interactions most likely results from the chatbot’s one-to-one interaction pattern (ie, involving 2 parties), as opposed to the one-to-many interactions provided by social media platforms (ie, where a single user can reach many other users).

Our research also showed that users were more likely to express their negative emotions when using social chatbots than on Twitter. All of the topics in the “Preventive behavior” and “Physical and psychological impact of COVID-19” themes, except for the “Avoidance” and “Vaccines”' topics, were more negative than positive. In contrast, earlier research found that COVID-19 topics’ sentiments on Twitter were either positive or neutral [2]. In particular, researchers found that the sentiment expressed on topics surrounding fear and masks was neutral or close to positive. In contrast, our study found that negative sentiments outweighed positive ones in both topics, with the “Fear” topic being associated with anxiety and sadness, and mask-related conversations being related to anger. This result suggests that people are more open to expressing negative emotions with a chatbot than on social media. We conjecture this is because virtual agents offer anonymity and privacy, allowing users to express themselves without fear of being judged [30]. Other studies have also identified that people are more open to discussing sensitive topics with virtual agents than humans [61]. These properties of chatbots suggest the potential for widespread AI-supported psychological support in times of crisis. For example, freely writing about one’s emotions and feelings (eg, expressive writing) has long been used as a treatment regimen to decrease depressive symptoms [62], and it has been shown to be effective in increasing COVID-19 resilience [63].

Regarding the sentiment analysis by country, we found that users in the United States shared the most negative and least positive sentiments, whereas users in the Philippines were the opposite. The attitudes of users in the United States toward “Preventive behaviors” topics were comparatively more negative than in other countries. This finding aligns with a previous Twitter study that reported users in the United States having strong negative feelings toward masks, quarantine, and social distancing. Additionally, we discovered that users in the United States were more negative when discussing the “Fear” and “Case and death counts” topics. These findings could be explained by the United States leading the world in coronavirus-related fatalities [64].

### Implications

Our findings have several ramifications for chatbot designers, health care professionals, and policymakers. Our findings suggest that there can be active collaboration between health organizations and chatbot platforms to address pandemic-related queries better and deliver accurate information to the general public. As identified previously [9], chatbots can deliver more concise information to users compared to social media or search engines. Designers can exploit this functionality to better inform users of critical and urgent health information. At the same time, there is also a concern that, as chatbots learn new information from users’ inputs, they could turn into a source of misinformation. In the past, major social platforms, such as Facebook, Instagram, YouTube, and Twitter, addressed misinformation by attaching warning labels, removing content, banning accounts, and connecting users with information from reputable sources [65]. Chatbots can adopt a similar strategy by providing links to trusted information sources, such as local CDCs and the WHO, satisfying users’ needs and disseminating accurate information.

However, adapting current chatbot strategies might not be enough to meet users’ needs. Most commercial chatbots are built for casual conversation, and the utterances they produce are based on the text corpora they were trained on. SimSimi is one such example, generating responses based on its existing data. New topics like COVID-19 cannot be easily handled. While some new chatbots employ search functionalities, such as Facebook’s Blender Bot 2.0 [7], they still suffer from various limitations [66]. For instance, Blender Bot 2.0 “still makes mistakes, like contradiction or repetition, and can *hallucinate* knowledge” [7]. More work is needed to address the limitations of large language models [67], including their ability to generate potentially harmful language and reinforce stereotypes [7,66]. In high-stakes scenarios like the COVID-19 pandemic, chatbots can be tailored to overcome these limitations and assist users in accessing crucial information rapidly.

Users’ tendency to view chatbots as social partners must be considered when designing chatbots that satisfy information needs. Our study suggests the importance of adding social functionalities to chatbots and offering critical public health information. Like in previous studies on conversational agents [5], users responded socially to a chatbot. For instance, users prayed for SimSimi’s safety and talked about their struggles due to COVID-19. Along with providing accurate information, social interaction will continue to be a key component of chatbots.

We also found that users were more likely to disclose their negative emotions toward COVID-19 with a chatbot than on Twitter. People’s tendency to open up when interacting with chatbots points to the possibility that these systems could develop into alternative tools for managing the depression and stress brought on by the pandemic, especially for replacing limited interactions due to lockdowns.

Finally, our research shows how chatbot data, like social media data, can be used to examine public perceptions and sentiments during a health crisis. People use the chatbot to express positive and negative emotions, and search for information like on any other digital platform. Rather than tracking individuals’ moods, the gross emotional state of users seen in chat conversations could be a barometer of the psychological impact of social upheaval. Chatbots could also get early feedback on policies that impact people’s everyday lives, such as wearing masks.

### Limitations and Future Work

Our study has several limitations. First, the data in this study are limited to users who accessed and used a particular chatbot. Given that the primary user group of the chatbot service studied is young adults [4], our findings may not be generalizable to other populations and age groups. Our study also focused on a particular social chatbot, and its findings might not be generalizable to other types of conversational agents.

Moreover, most research on chatbots for COVID-19, including this study, has been limited to English data. In particular, we analyzed English conversations from countries whose native language is not English (eg, Malaysia and the Philippines). Our results might thus be specific to users who are proficient in English and may not be generalizable to all users and citizens in Malaysia and the Philippines. Therefore, future studies could consider multilingual topic modeling and sentiment analysis.

Furthermore, due to the limitations of existing computational approaches, we focused on positive and negative sentiments rather than neutral ones in this study. However, studying neutral sentiments could be an interesting future line of work.

Finally, we assumed that each conversation block consisting of 3 consecutive utterances could be classified into a single topic, consistent with conventional topic modeling. However, users’ conversations with chatbots may cut through various subjects at the same time. Future work could explore how coronavirus-related topics intersect by using multiclass labels.

### Conclusions

This study was the first to analyze chatbot-user conversations from a live system over 2 years in the context of the COVID-19 pandemic. We examined user conversations about COVID-19 with a commercial social chatbot using NLP tools and found 18 topics that were categorized into 5 overarching themes. Our findings demonstrate how people used the chatbot to seek information about the global health crisis, despite the chatbot not being designed as a resource for such information. Users’ expectations of chatbots to simultaneously play particular social roles, like engaging in small talk, show that these conversational agents may need to be created with various social functions. The sentiments expressed by users were then examined in relation to how these topics were discussed, showing that people were more likely to engage in emotional conversations with a chatbot than on social media.

Chatbot data can be used to better understand people's emotional and informational needs during crises like the COVID-19 pandemic. Instead of creating responses based on pretrained text, virtual social agent designers can consider the informational needs of people looking for the latest and most accurate information. Initiatives like Facebook’s Blender Bot 2.0 [66] can search the internet for information not covered during the training processes, and such search functionality will be useful in meeting users’ information needs. Additionally, given that people tend to be more open to chatbots when sharing negative emotions [61], chatbots can play an increasing role in meeting the emotional needs of users and helping alleviate depressive moods.

## Appendix

Multimedia Appendix 1 The average percentage of positive; negative; and anger-, anxiety-, and sadness-related words by 5 countries in COVID-19–related conversations according to the Linguistic Inquiry and Word Count dictionary. Error bars indicate standard errors. CA: Canada, GB: United Kingdom; MY: Malaysia; PH: Philippines; US: United States.

Multimedia Appendix 2 The average percentage of positive and negative words in COVID-19–related chat sessions by topic and country, according to the Linguistic Inquiry and Word Count dictionary. Error bars indicate standard errors.

## Abbreviations

AI: artificial intelligence
API: application programming interface
CDC: Centers for Disease Control and Prevention
LDA: Latent Dirichlet Allocation
LIWC: Linguistic Inquiry and Word Count
NLP: natural language processing
WHO: World Health Organization
