# Supplementary figures and images for: User-Chatbot Conversations During the COVID-19 Pandemic: Study Based on Topic Modeling and Sentiment Analysis
Source: J Med Internet Res. 2023 Jan 27;25:e40922. doi: 10.2196/40922 (PMC9885754; doi:10.2196/40922)

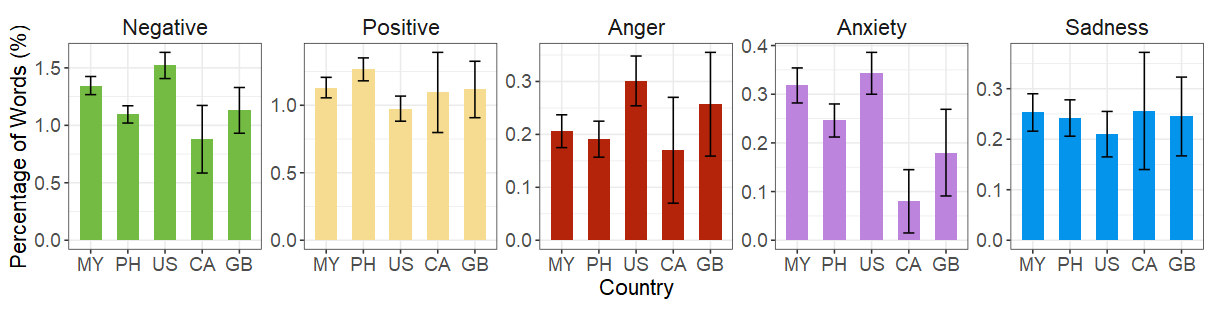

Supplement: Multimedia Appendix 1 [file jmir_v25i1e40922_app1.png]

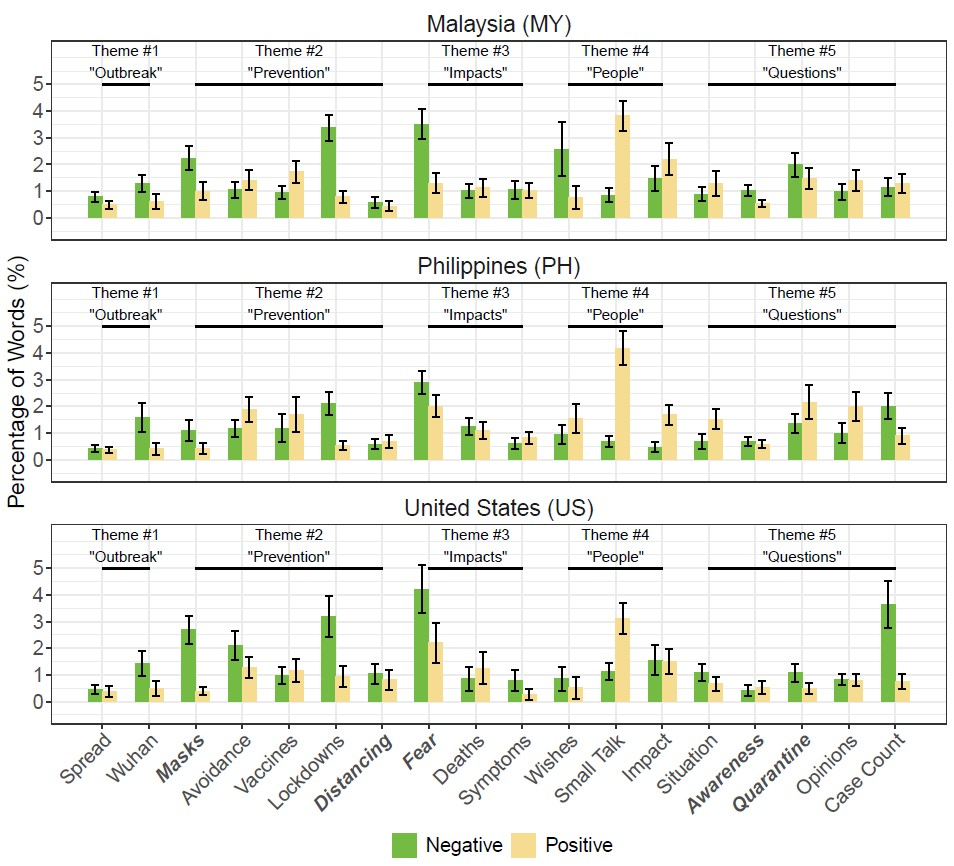

Supplement: Multimedia Appendix 2 [file jmir_v25i1e40922_app2.png]
